# Mediastinal lipoma in an extrapulmonary tuberculosis patient

**DOI:** 10.11604/pamj.2025.52.46.48770

**Published:** 2025-09-29

**Authors:** Amit Toshniwal, Manish Meshram

**Affiliations:** 1 Department of Respiratory Medicine, Datta Meghe Institute of Higher Education and Research, Wardha, Maharashtra, India,; 2 Department of Respiratory Medicine, Government Hospital, Gadchiroli, Maharashtra, India

**Keywords:** Lung cancer, cardiac lipoma, tuberculosis

## Image in medicine

A 76-year-old man presented with progressive breathlessness, dry cough, loss of weight, and vague chest pain for three weeks. He denied a history of fever. Clinical examination revealed decreased breath sounds and dullness to percussion over the right hemithorax infra-scapular area. Chest radiography revealed right-sided pleural effusion with abnormal opacity near the mediastinum (panel A). A contrast-enhanced computed tomography (CECT) thorax revealed right-sided pleural effusion with a well-encapsulated, homogenous fat-density lesion in the anterior mediastinum of approximately 70 x 55 mm, confirming mediastinal lipoma (panel B). There were no compressive symptoms or signs of malignancy. Diagnostic thoracocentesis revealed straw-coloured exudative pleural fluid with elevated adenosine deaminase (ADA), confirming extrapulmonary tuberculosis (TB). The patient was initiated on antitubercular therapy and supportive care. Given the benign nature and asymptomatic status of lipoma, conservative management with follow-up was advised. The co-detection of mediastinal mass and extrapulmonary TB raises important considerations in differential diagnosis, surveillance, and long-term follow-up, especially in elderly patients with weakened immunity or other predisposing conditions.

**Figure 1:**
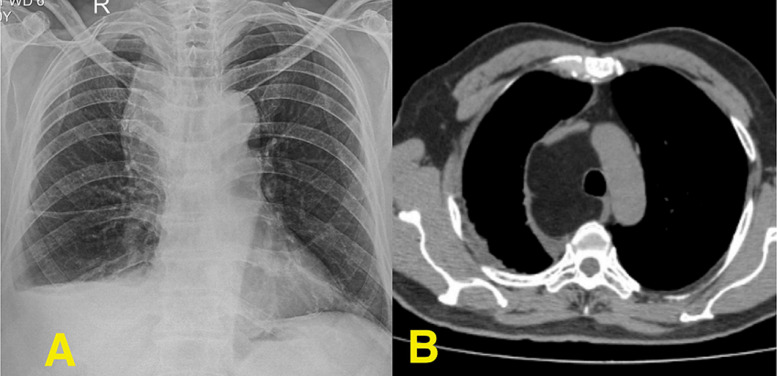
A) chest X-ray showing right pleural effusion with opacity in mediastinum, B) axial section of CECT thorax showing well-encapsulated, homogenous fat-density lesion in the mediastinum

